# Supplementary material for: Synergistic effects of Clostridium butyricum and Akkermansia muciniphila-derived postbiotics ameliorate DSS-induced colitis and associated tumorigenesis through immunomodulation and microbiota regulation in mice
Source: mSystems. 2025 Dec 29;11(2):e00689-25. doi: 10.1128/msystems.00689-25 (PMC12911357; doi:10.1128/msystems.00689-25)
Supplement: Supplemental figures — Original Western blot images. [file msystems.00689-25-s0001.docx]

**Original Western Blot Images**

**Figure S1**

**Western Blot detection of the key subunit P65 protein in the NF-κB signaling pathway**


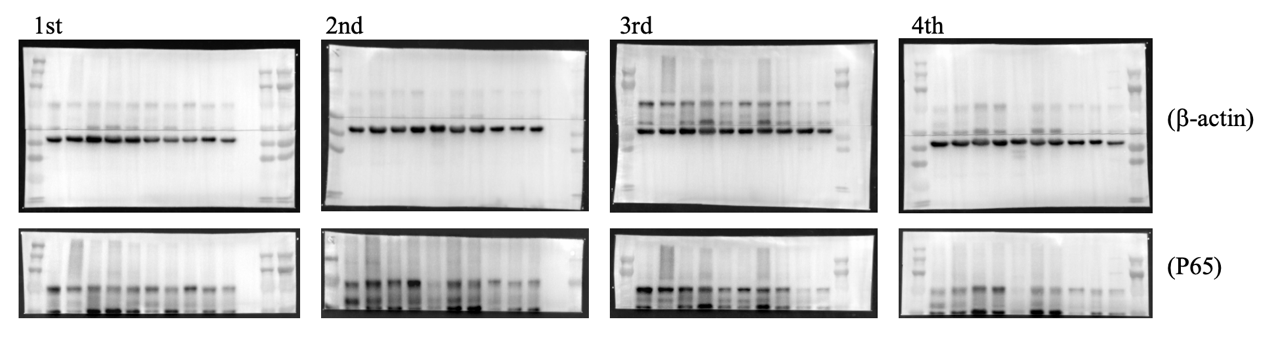


*All measurements were performed with a minimum of three technical replicates.

**Figure S2**

**Western Blot detection of the key subunit TLR4 protein in the NF-κB signaling pathway**

**
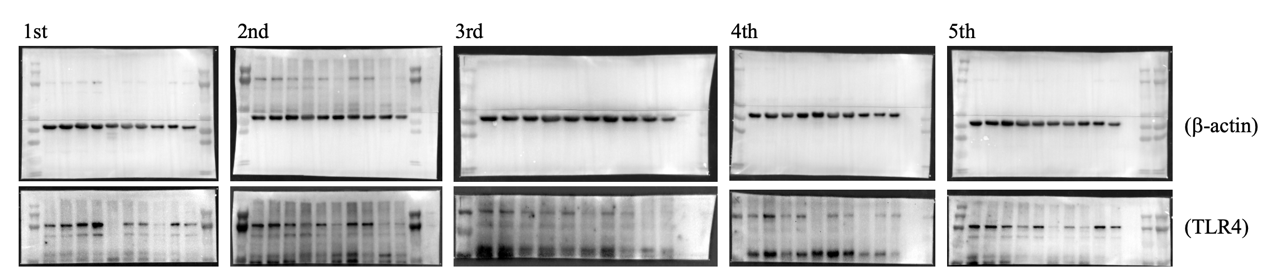
**

*All measurements were performed with a minimum of three technical replicates.
